# CRISPR/Cas9 mediated targeting of multiple genes in *Dictyostelium*

**DOI:** 10.1038/s41598-018-26756-z

**Published:** 2018-05-31

**Authors:** Ryoya Sekine, Takefumi Kawata, Tetsuya Muramoto

**Affiliations:** Department of Biology, Faculty of Science, Toho University, 2-2-1 Miyama, Funabashi, Chiba 274-8510 Japan

## Abstract

CRISPR/Cas9 has emerged in various organisms as a powerful technology for targeted gene knockout; however, no reports of editing the *Dictyostelium* genome efficiently using this system are available. We describe here the application of CRISPR/Cas9-mediated gene modification in *Dictyostelium*. The endogenous tRNA-processing system for expressing sgRNA was approximately 10 times more effective than the commonly used U6 promoter. The resulting sgRNA affected the sub-nuclear localisation of Cas9, indicating that the expression level of sgRNA was sufficiently high to form Cas9 and sgRNA complexes within the nucleus. The all-in-one vector containing Cas9 and sgRNA was transiently expressed to generate mutants in five PI3K genes. Mutation detective PCR revealed the mutagenesis frequency of the individual genes to be between 72.9% and 100%. We confirmed that all five targeting loci in the four independent clones had insertion/deletion mutations in their target sites. Thus, we show that the CRISPR/Cas9 system can be used in *Dictyostelium* cells to enable efficient genome editing of multiple genes. Since this system utilises transient expression of the all-in-one vector, it has the advantage that the drug resistance cassette is not integrated into the genome and simple vector construction, involving annealing two oligo-DNAs.

## Introduction

The simple amoeboid eukaryote *Dictyostelium discoideum* is a model system, that is used to study several biological processes such as growth, macropinocytosis, cell motility, chemotaxis, and signal transduction during development^1^. It possesses homologous of genes in complex eukaryotes related to these processes, which are missing in the well-established model system of *Saccharomyces cerevisiae*^2^. Several attempts to manipulate the gene function, including homologous recombination for gene disruption^3,4^, knock-in and RNA interference (RNAi)^5^, have been widely used to analyse these functions. Due to the haploid genome, simple and highly efficient methods are possible. Using the homologous recombination technique, a DNA template typically over 500 bp homology sequences, is designed to recombine at the genomic locus of interest.

Because many of these genes reveal overlapping functions, there is also a high demand for the generation of multiple gene knockouts within a single cell. Although blasticidin is an efficient selectable marker to produce gene knockout by homologous recombination, the limitation on the number of selectable markers restricts the generation of multiple gene deletions. Targeting at a single locus is less efficient because G418, another highly used marker, is present in multiple copies correlated with the concentration of the drug^6^. The Cre-*loxP* system for multiple gene knockouts was developed to overcome these limitations^7,8^. Indeed, a quintuple PI3K knockout mutant was created, by which five genes were sequentially knocked out by recycling the blasticidin marker^9^; however, as it requires a minimum of several months to generate the quintuple mutant, the long-term cell culture might induce unexpected mutations within the genome.

Multiple gene targeting has proved successful in applying the genome editing technologies in various model systems^10–13^. One of the powerful genome editing tools, the clustered regularly interspaced short palindromic repeats (CRISPR) associated protein-9 nuclease (Cas9) mediated genome editing, has been widely used for functional genomic studies. The Cas9/sgRNA complex can efficiently generate site-specific double-strand break (DSB), resulting in the activation of the DSB repair pathway, as demonstrated by previous studies in several organisms. The DSB can be then repaired by non-homologous end-joining (NHEJ), which often leads to random deletion at the sites. In *Dictyostelium*, the repair of the DSB is well studied, and several proteins essential for NHEJ, such as Ku and DNA-PKcs, have been analysed^14,15^. Nevertheless, to date, there are no reports of successful gene disruption using the CRISPR/Cas9 system in *Dictyostelium*. One reason is that, in contrast to mammalian cells and tissues, homologous recombination in *Dictyostelium* is extremely efficient and accurate. There exist, however, many cases in which the specificity of homologous sequences is lower at the gene of interest. In the CRISPR/Cas9 system, because a ~20 nucleotide target sequence is used to achieve site-specific DSB, these difficulties can be avoided in some cases. Furthermore, the simple procedure of CRISPR/Cas9 has created a possibility of applying transcriptional activation/repression, epigenetic modification, genomic imaging and the targeting of multiple genes^16–18^.

A chimeric single-guide RNA (sgRNA), the target sequence, directs the Cas9 nuclease to a specific cleavage site. Although the Cas9 and sgRNA complex may bind any genome sequence, the only requirement for the selection of the target site is the presence of a specific protospacer adjacent motif (PAM) sequence of 5′-NGG-3′, which appears at a relatively high frequency within the gene region, even in the AT-rich *Dictyostelium* genome. The most important determinants in the success of targeting are 12-nt upstream of the PAM. A mismatch in this region leads to a considerable reduction in the cleavage activity^19^. Additionally, a TTTT stretch, known as a termination signal for RNA polymerase III, should be avoided in the sgRNA sequence. In the generally used CRISPR/Cas9 systems, the sgRNA expression is derived by RNA polymerase III-dependent promoters such as U6. The *Dictyostelium* U6 snRNA is thought to be transcribed by RNA polymerase III, as it lacks the trimethylated 5′ cap for U6 as is observed in other organisms; however, direct evidence remains sparse^20^. Moreover, the use of the U6 promoter requires a ‘G’ residue as the first base of the sgRNA to initiate transcription, which restricts the number of appropriate target sequences. It is thus desirable to use an expression system without this sequence limitation. Because RNA polymerase III synthesises all tRNAs in the eukaryotes, the endogenous tRNA-processing system could be used to express sgRNA in *Dictyostelium*. The insertion of sgRNA between tRNA sequences causes RNase P and RNase Z to recognise the cloverleaf structure and cleave at the 5′ and 3′ ends of tRNA, resulting in the production of mature sgRNAs^21^.

Here, we successfully developed a highly efficient and simple CRISPR/Cas9 mediated simultaneous multiplex genome editing system by a single plasmid in *Dictyostelium*. Using this system, we generated *pikA*, *pikB*, *pikC*, *pikF*, and *pikG* quintuple genome modified cells. Because the mutagenesis is mediated by transient expression of the all-in-one vector, any drug resistance cassette can be used for further analysis. Our data suggest that this system is a powerful tool for the analysis of gene function using multiplex genome modifications in *Dictyostelium*.

## Results

### Construction of CRISPR/Cas9 expression system

**Figure 1:**
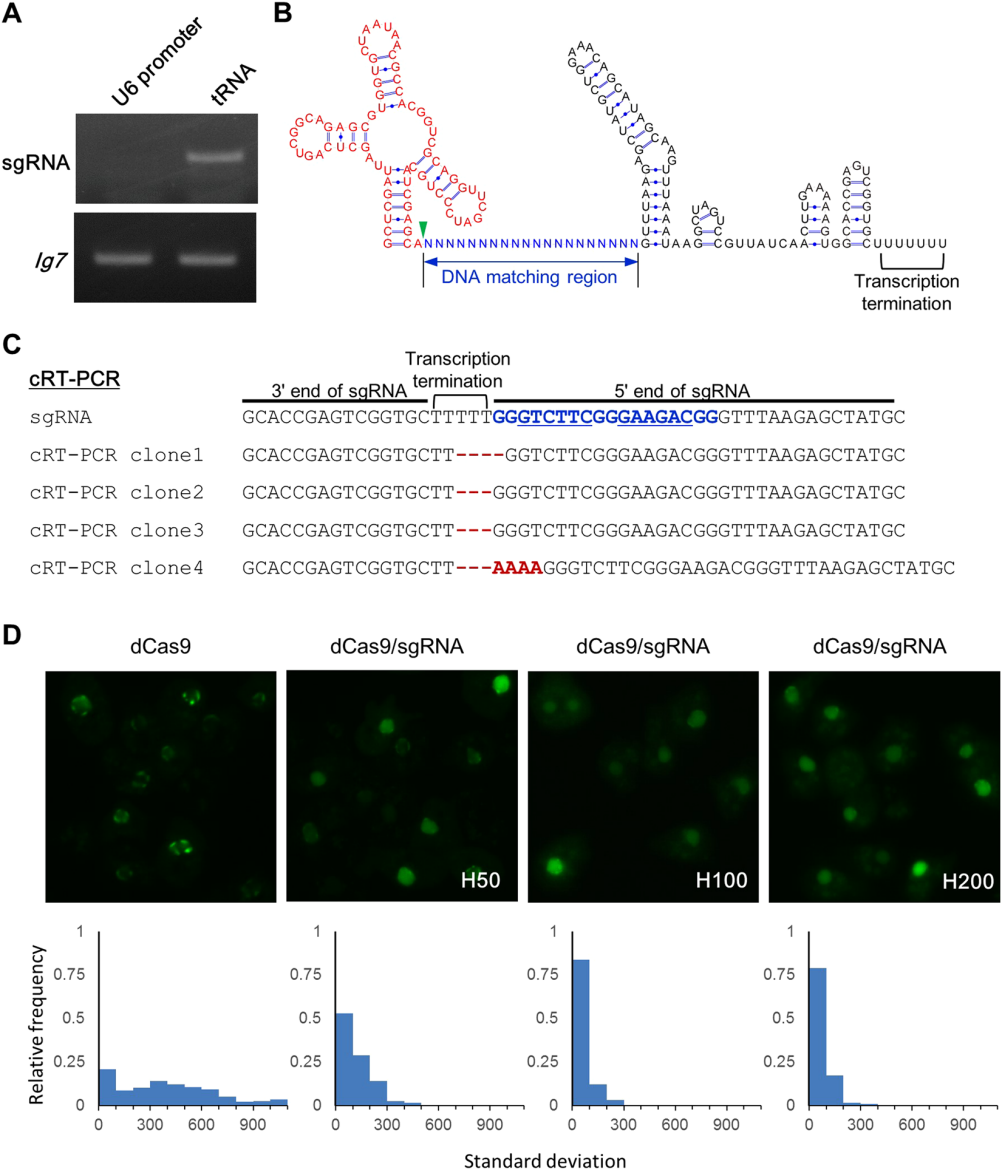
Endogenous tRNA system for expressing sgRNAs. (**A**) Comparison of sgRNA expression using different promoters. RT-PCR of sgRNA is presented. The lower panel reveals Ig7 as an internal control. Gel images were cropped, but no other bands were present. (**B**) Predicted secondary structure of tRNA-sgRNA. The nucleotides of isoleucine tRNA are indicated in red, and the DNA matching region is presented in blue. The green arrowhead indicates the tRNA cleavage site to release sgRNA. (**C**) Sequence analysis of the tRNA-sgRNA junction by cRT-PCR. Sequences of four independent clones are presented. As empty sgRNA vector was sequenced, the DNA matching region shown in blue contains two BpiI sites (underlined) to insert a pair of annealed oligonucleotides. The extra nucleotide at 5′ region is given in red. (**D**) The sub-nuclear localisation of dCas9-GFP. All the dCas9-expressing transformants were maintained with 60 µg/ml G418 for a few days before imaging. Cells expressing sgRNA were cultured with hygromycin B at 50, 100 and 200 µg/ml, respectively. Graphs reveal a histogram of standard deviation (SD) of fluorescence distribution within the nucleus. Lower SD indicates a uniform distribution of dCas9-GFP in the nucleus.

CRISPR/Cas9 targeting requires molecular cloning of a sgRNA that contains a target-specific sequence. We used Flip and Extension (F + E) modified sgRNA because the modified hairpin structure was designed to increase its transcription level and to enhance its assembly with the Cas9^22^. Previous work has demonstrated that high levels of sgRNA expression increased the gene targeting efficiency in human cells^23^. To compare the expression efficiency of the sgRNA in *Dictyostelium* under the control of a different promoter, we constructed two sgRNA expression vectors derived from the U6 promoter or isoleucine tRNA. The expression level of sgRNA derived from tRNA was approximately 10 times higher than that of the U6 promoter (Fig. 1A). Through the insertion of sgRNAs after tRNAs, endogenous tRNA processing machinery naturally cleaves sgRNAs using RNase P and RNase Z (Fig. 1B). We performed circularised RT-PCR (cRT-PCR) to analyse the end sequences of mature sgRNAs. The basic principle of cRT-PCR is that the RNAs are circularised by RNA ligase before cDNA synthesis and PCR amplification of the end sequences. All gRNA transcripts were cleaved at the tRNA-sgRNA junction and a half of the clones were cleaved precisely without the addition of extra nucleotides (Fig. 1C). The 3′ end of mature gRNAs were terminated within a Pol III terminator of a T stretch.

In the CRISPR/Cas9 system, the assembly of Cas9 and sgRNA complexes is essential for binding and cleaving DNA *in vivo*. To examine if the complexes were formed efficiently, we observed the localisation changes of Cas9 using dCas9-GFP expressing cell lines. Sub-nuclear relocalisation of dCas9 from the nucleolus to the nucleoplasm is known to depend on the expression level of sgRNA^22^. Although dCas9 was found as foci at the nuclear periphery (presumably nucleoli) in the cell line expressing dCas9-GFP without sgRNA, its localisation changed to the nucleoplasm when sgRNAs were expressed in the cells (Fig. 1D). This effect was strongly observed in the cells, which were maintained in a high concentration of hygromycin B, a higher concentration of which is thought to increase the expression level through the extrachromosomal vector. These results were consistent with the findings in mammalian cells. These observations imply that the expression level of sgRNA through the tRNA expression system was enough to form Cas9 and sgRNA complexes.

### Cas9/sgRNA mediated deletion in tdTomato knock-in cells

**Figure 2:**
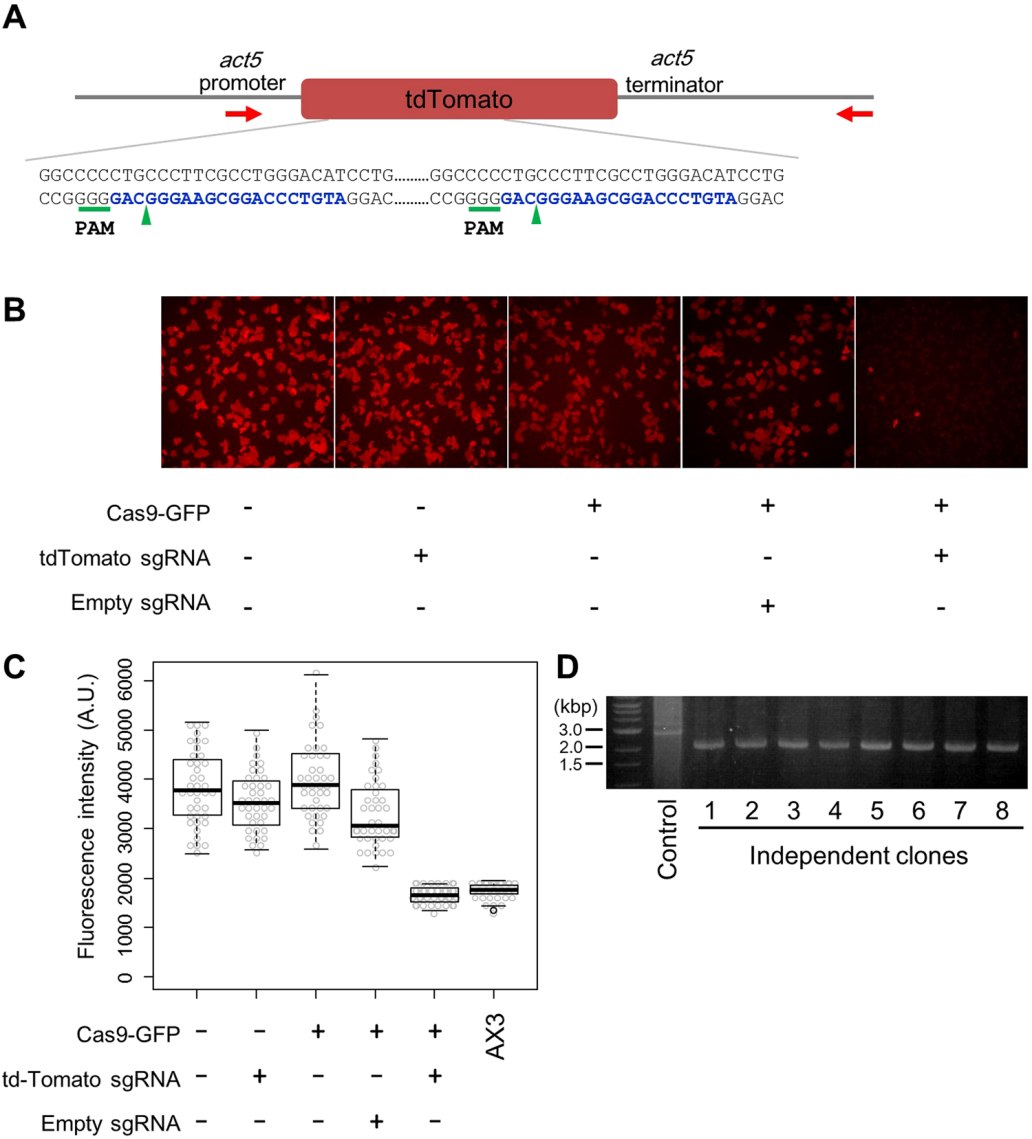
CRISPR/Cas9-mediated deletion in tdTomato knock-in cells. (**A**) Schematic diagram of the target sites (blue) in tdTomato knock-in cells and primers (red arrow) for PCR assay. Green arrowheads indicate predicted cleavage site by Cas9. (**B**) Fluorescence microscopic observation of the expression level of tdTomato. The Cas9-GFP, tdTomato sgRNA or empty sgRNA expression construct was introduced into the tdTomato-expressing cells, and the resulting fluorescence was imaged under identical conditions. Red fluorescence images of a representative area are shown. (**C**) Intracellular fluorescence intensity levels in different CRISPR conditions. Each dot represents an individual cell. AX3 reveals the fluorescence level of parental strain without tdTomato. (**D**) PCR amplification using primers flanking the target site. Lane 1 presents the tdTomato knock-in cell as a control, whereas rest of the lanes represent eight individual clones expressing both Cas9 and sgRNA. Gel image was cropped, but no other bands were present.

To evaluate whether the Cas9/sgRNA system causes mutation at the target site in *Dictyostelium*, we generated cells in which the gene for tdTomato was knocked-in to the *act5* gene locus, and measured the disappearance of red fluorescence due to the Cas9/sgRNA mediated mutation in the gene by fluorescence microscopy. Since the sequence for the tdTomato contains tandem repeats, a genomic deletion of approximately 700 bp can be generated using a single sgRNA for targeting (Fig. 2A). Upon co-transformation of *Dictyostelium* cells with the stable expression vectors for both Cas9 and tdTomato sgRNA, the red fluorescence intensity of most of the cells decreased to wild type (AX3) level (Fig. 2B,C). The targeting efficiency at a single cell level was 99.4% (n = 1618) compared with a negative control such as non-targeting sgRNA or no Cas9 expression. In contrast, the cells with tdTomato sgRNA alone, Cas9 alone or empty sgRNA expression vector without targeting sequence revealed no visible difference from parental tdTomato knock-in cells. To confirm the targeted gene deletion, we extracted the genomic DNA and amplified it by PCR, and a 700 bp gene deletion was confirmed in all 8 randomly selected clones (Fig. 2D). These results indicated that the sgRNA recruited Cas9 endonuclease to the appropriate target site and modified the genome with high efficiency.

### Transient expression mediated gene modification in endogenous gene

**Figure 3:**
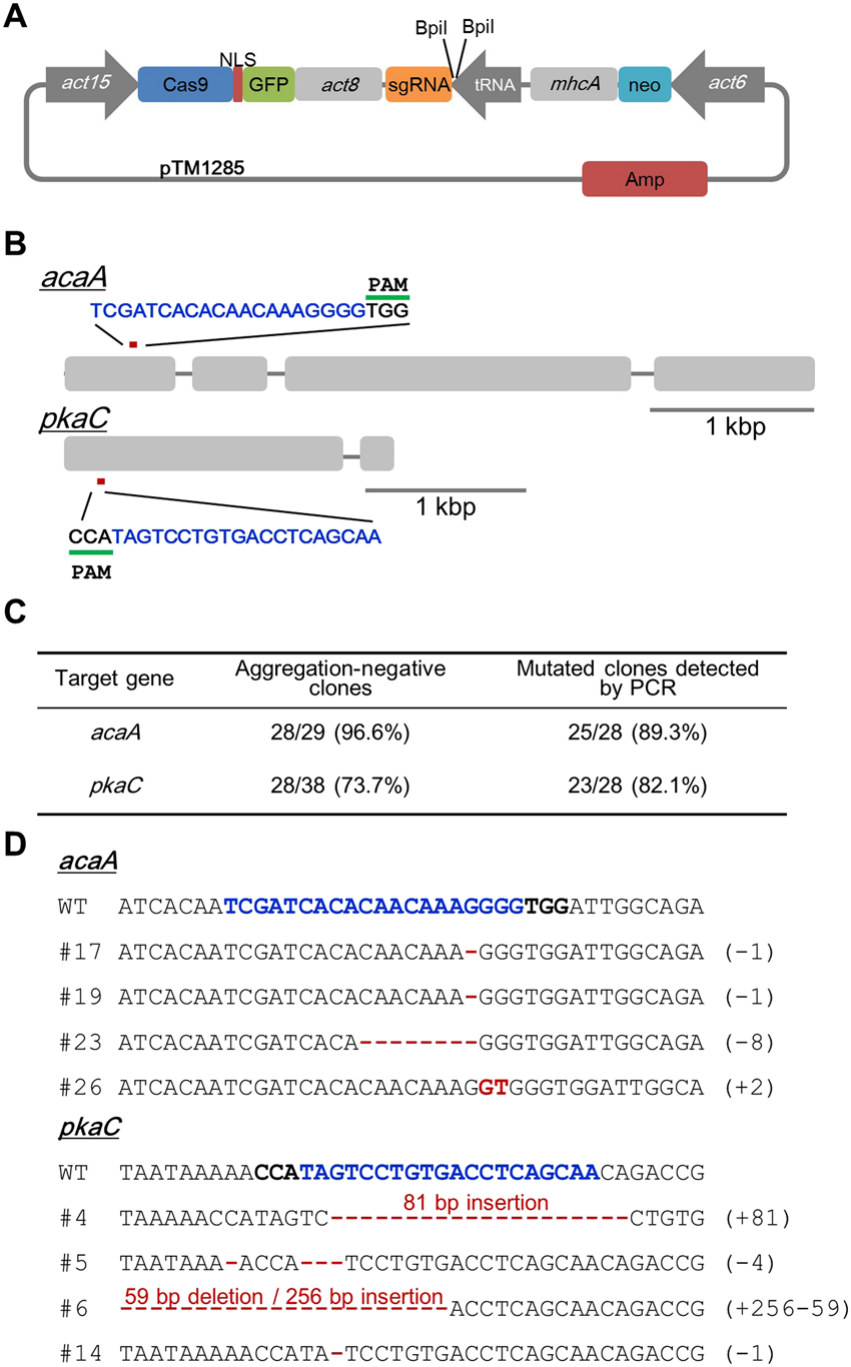
Targeting of two developmental genes using CRISPR. (**A**) Schematic overview of the all-in-one CRISPR/Cas9 vector, pTM1285. A pair of annealed oligonucleotides is assembled into the all-in-one vector using the Golden Gate assembly method. Amp, ampicillin resistance gene; *act15*, *act15* promoter; *act8*, *act8* terminator; tRNA, isoleucine tRNA, *mhcA*, *mhcA* terminator; neo, neomycin resistance gene; *act6*, *act6* promoter. (**B**) Schematic view of sgRNA targeting locus of the *acaA* and *pkaC* genes. The blue sequences indicate the target sites of the sgRNA. (**C**) Frequency of aggregation-negative and mutated clones. (**D**) Wild-type sequence for *acaA* and *pkaC* and sequences derived from 4 independent positive clones by mutation detective PCR are presented. The target sequence is shown in blue, and mutations are shown in red. Numbers in parentheses indicate the number of modified nucleotides.

To generate accurate and straightforward genome editing system in *Dictyostelium*, we developed a transient expression system. Constitutive expression of Cas9 and sgRNA, which were used in the targeting of the tdTomato, requires drug selection and increases the risk of off-target activity, which makes insertions or deletions other than the intended on-target sequence. Transient expression is widely used in Cre recombinase-mediated recombination in *Dictyostelium*. Indeed, it is reported that transient expression with temporary G418 selection removed the Bsr cassette with a high frequency^7^. Since the transient expression vector for Cre recombinase comprised the Cre cassette including promoter and terminator and the G418 resistance cassette without a sequence for plasmid replication in *Dictyostelium*, we generated an all-in-one sgRNA and Cas9 expression vector including the G418 resistance cassette (Fig. 3A) and then evaluated its efficiency for gene modification in the endogenous genes.

Two endogenous genes, *acaA* and *pkaC*, were selected as target genes (Fig. 3B). As the knockout mutants were demonstrated to have a defect in cell aggregation^24,25^, the phenotype of individual clones was assessed as either aggregation-positive or aggregation-negative. Because almost all cells were able to recover after the temporary G418 selection, we obtained more than 100,000 transformants from one transformation. For the assessment, we selected independent clones randomly, and the relative number of aggregation-negative clones of *pkaC* and *acaA* were 96.6 and 73.7%, respectively. Using mutation detective PCR screening, we determined that more than 80% of the aggregation-negative clones were positive for targeting (Figs 3C and S2). All of the aggregation-negative clones were unable to grow in G418, indicating that the all-in-one vector was potentially not integrated into the *Dictyostelium* genome, but the possibility that fragments of the plasmids are retained cannot be eliminated. This observation was also confirmed by fluorescence microscopy, where no aggregation-negative clone revealed a Cas9-GFP signal. To analyse indels at the *pkaC* and *acaA* gene loci generated by transient sgRNA and Cas9 expression, we amplified the targeting regions by PCR. The results of sequencing revealed that all the analysed clones caused mutations in the target locus (Fig. 3D). These data indicate that we successfully generated a single gene knockout using a transient expression system without integrating a drug-resistant gene.

### Targeting of Multiple genes in endogenous gene

**Figure 4:**
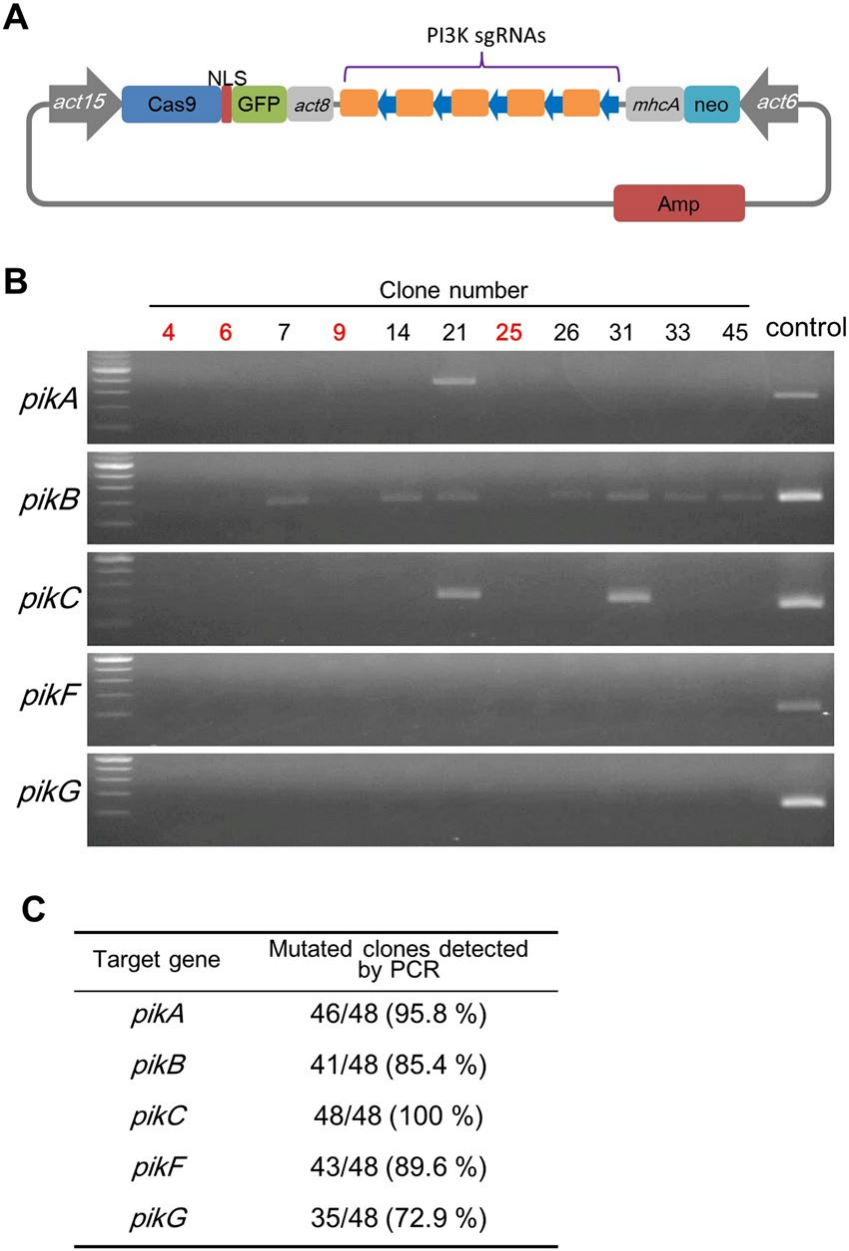
Multiplex genome editing with CRISPR/Cas9. (**A**) Schematic depiction for simultaneously targeting of multiple genes. Five sgRNAs were assembled using Golden Gate system and inserted into the multiplex expression vector. Blue arrows indicate tRNA and orange boxes indicate each sgRNA. (**B**) Identification of mutations in the targeting locus of PI3K genes. Clones revealing low amplification efficiency of the PCR products compared to the control were candidate mutants. Clone numbers presented in red were used for further sequence analysis. Parental strain AX2 was used as a control. The sizes of the PCR products were 217 bp, 167 bp, 184 bp, 126 bp and 102 bp, respectively. Gel images were cropped, but no other bands were present. (**C**) Summary of candidate mutants mediated by multiplex CRISPR/Cas9.

Manipulation of multiple genes by conventional homologous recombination using the Cre-*loxP* system in *Dictyostelium* takes several months. To simplify the manipulation of multiple genes, we examined whether the transient expression of sgRNA/Cas9 can be used for targeting of multiple genes. We prepared a new multiplex sgRNA vector in order to express multiple sgRNAs from one expression vector. We designed sgRNAs for targeting five PI3K genes whose genes had already been knocked out by homologous recombination^9^ and inserted them into the multiplex vector (Fig. 4A). To combine the repetitive tRNA-gRNA cassette, we used the Golden Gate assembly strategy as described in the Materials and Methods section (Supplementary Fig. S3). Using this method, it is possible to target up to 20 genes with one vector.

**Figure 5:**
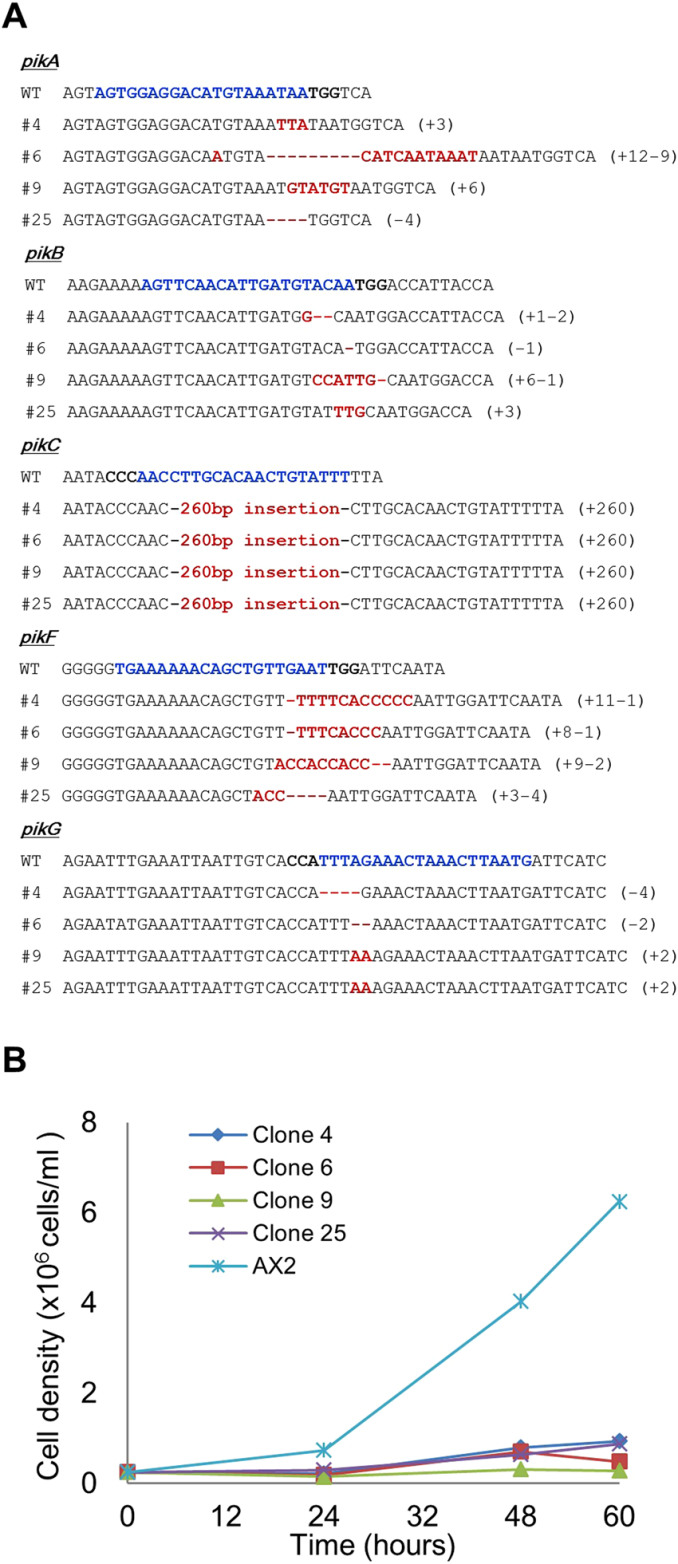
Genomic deletions and insertions in the targeting loci of PI3K genes. (**A**) The sequencing results of the targeting regions. Wild-type (WT) sequences of five PI3K genes (top) and sequences of 4 independent mutants are presented. The target sequence is in blue, and mutations are in red. Numbers in parentheses indicate the number of modified nucleotides. (**B**) Growth curve of the CRISPR mutants. Parental strain AX2 was used as a control.

We then introduced the multiplex transient expression vector for five PI3K genes into *Dictyostelium* cells. After the transient expression, the cells were allowed to recover on the SM agar plates with a bacterial lawn after which we amplified the target region by PCR (Fig. 4B). The mutation detective PCR revealed that the mutagenesis frequency of the individual genes was between 72.9 and 100% (Fig. 4C). We used four of the mutated clones for further sequencing analysis. Based on the sequencing results, all the five targeted loci in the four independent clones had indel mutations in the target sites, including both insertion and deletion (Fig. 5A); however, we noted a few cases where 3 or 6 nucleotides were inserted at the genomic loci, resulting in the insertion of 1 or 2 amino acids in the protein, which presumably retained some function. These mutations may be enriched due to growth of the cells in axenic medium during transfection, since the knockout of all five PI3K genes results in very poor growth in liquid medium^9^. Even so, we obtained quintuple genome modified cells by transient expression of CRISPR/Cas9, and the multiple mutants grew poorly in axenic medium (Fig. 5B), as expected^9,26^.

## Discussion

In this report, we have demonstrated that the CRISPR/Cas9 system can be used in *Dictyostelium* cells to enable efficient genome editing of multiple genes. This method has three distinct advantages over gene targeting techniques using conventional homologous recombination. First, since the all-in-one vector is transiently expressed, the drug resistance marker is not integrated into the genome, and in subsequent experiments, gene manipulation using an arbitrary drug marker can be performed again. Second, the preparation of a vector expressing the target sequence is convenient and user-friendly. The preparation of a general gene disruption construct requires several steps of cloning. In contrast, here a fragment of annealed two 24-nt oligos is cloned into the all-in-one vector by a Golden Gate assembly using DNA ligase and a type IIS restriction enzyme. Third, the time required for disruption of multiple genes is significantly shortened. At least 6 months are required to disrupt all five genes, such as PI3K 1–5 using homologous recombination. Using the CRISPR/Cas9 system, it is possible to reduce the accumulation of random mutations caused by long-term cell culture.

In various organisms, the U6 promoter is used to express sgRNA; however, in *Dictyostelium*, we found that the expression level of sgRNA under the control of U6 promoter was lower than that achieved using tRNA. Regions required for efficient transcription *in vivo* are widely reported to be missing within the U6 gene in mammals, whereas in yeast, the regions were identified both within the gene region and downstream of the RNA coding region^27^. Therefore, the reason for the low transcriptional activity under the control of the U6 promoter in *Dictyostelium* is suggested to be similar to that of yeast, where the region involved in the transcriptional regulation is included within the gene. This hypothesis is also supported by the fact that the expression level of sgRNA is higher under the control of the U6 region including both the U6 promoter and the gene region (data not shown).

Although CRISPR/Cas9 is a simple and highly efficient system as compared with conventional homologous recombination, it is known that CRISPR can sometimes make insertions or deletions in locations other than the target sequence, which is called off-target activity. In some cases, analysis by deep sequencing was performed on CRISPR-mediated mammalian cultured cells to investigate the off-target effects comprehensively^28^; however, it is hard to say that it is a cost-effective method. To minimise the off-target effect, Cas9 nickase, which is capable of introducing a single-strand nick, is useful^29^. Cas9 nickase and a pair of sgRNAs that bind to the opposite strand of DNA generate a site-specific DSB and are used to improve the specificity of the genome editing. Web tools such as ‘COSMID’ and ‘Cas-OFFinder’ are used to minimise the effect because they predict potential off-target sites for candidate sgRNAs^30,31^. Therefore, in the design of the target sequence, the possibility of off-target activity was significantly reduced by selecting a sequence in which the next most related sequence in the *Dictyostelium* genome had at least 5 mismatches within 12 nucleotides of the PAM sequence. Additionally, since we used a transient expression system to drive the CRISPR system, genome modification did not occur continuously, and the risk of off-target activities was reduced; however, it is assumed that the off-target effects still occur fundamentally at low frequencies, so it is essential to compare and analyse several CRISPR-mediated clones.

Although the design of highly specific target sequences is essential for reducing the off-target activity, depending on the gene, it may be difficult to find the appropriate sgRNA because the GC-rich NGG PAM sequence does not frequently appear in the AT-rich *Dictyostelium* genome. Among the 7 endogenous genes used in this paper, approximately 3 candidate sgRNAs are predicted within the first 1000 bp. As a solution to this problem, a class 2 CRISPR, recognising the T-rich TTTV PAM sequence (TTTA, TTTC, and TTTG) and then inducing DNA cleavage, is useful^32^. Compared to the case where NGG was used as the PAM sequence, when TTTV was used as a PAM sequence, the potential choice of the target sequence to the PI3K genes increased by 50%. A poly-T stretch should be avoided from a sgRNA sequence because a TTTT sequence is known as a termination signal for RNA polymerase III. Based on the limitations of target sequences, the designing sgRNAs for knock-in is more difficult than for knock-out.

In the case of gene knock-out using conventional homologous recombination, genes essential for cell growth cannot be disrupted. In our experiments, we successfully knocked out the *acaA* and *pkaC* genes affecting cell aggregation, while the PI3K knock-out strains possessed one or two amino acid insertions in one of the five genes. Because PI3K knock-out strains reveal slow growth in the axenic medium^9,26^, indel mutations without significant defect were enriched during cell growth in the medium. The maintenance of cells with bacteria rather than the axenic medium is one solution to this problem, made possible by recent improvements in methods for selecting transformants on bacteria^33^. Other solutions are targeting the gene again with CRISPR/Cas9, or creating deletion in the target gene region by designing two target sequences for one gene. A deletion of 107 bp within the *pkaC* gene was produced using two different sgRNAs (data not shown), confirming that this method can be used for making targeted deletion in genes. Thus the creation of deletion should be considered to generate complete loss of function mutants. We also showed that targeting of multiple genes other than PI3K genes simultaneously by introducing mixture of different sgRNA expression vectors (data not shown). Due to the ease of generating sgRNA, a genome-wide library of sgRNAs provides an opportunity to perform high-throughput genetic screening in *Dictyostelium*. Its application has been already reported in mammalian systems^34,35^. Regarding the inserted sequence, in *pikF*, a sequence of 260 nucleotides was inserted in all four independently isolated clones. These insertional nucleotides were originally from a CRISPR vector; however, there is no homology between the genome region and the inserted sequence. This result suggests that it is also possible to generate knock-in cell lines using NHEJ-mediated targeted integration, HITI^36^.

In general, *Dictyostelium* has a mature technology for creating knock-outs and knock-ins using homologous recombination. CRISPR/Cas9 has the potential to add another dimension to this technology. In particular, it is very quick and efficient. Thus, the targeting of single and multiple genes can be completed within 7 days–10 days. Since multiple clones need to be analysed to avoid off-target effects, in the situation where this is difficult because of complicated steps or experimental cost, the conventional method apparently has advantages. In this research, we used *D. discoideum*, the most common species for research, but there are about 100 other species of Dictyostelia. These are divided into four groups, and *D. discoideum* is classified into recently branched group 4^37^. In many other species, apart from *Polysphondylium pallidum*, it is hard to generate gene knock-out since the efficient method of gene disruption by homologous recombination has not been established. One of the causes is that blasticidin S, G418 and hygromycin B, which are widely used drugs in *D. discoideum* are less effective or have no effect on other Dictyostelia species. In our system, a transient expression system is used, and continuous drug selection is not necessary. Furthermore, since genome editing was confirmed for more than 50% of clones obtained from transient selection with G418, our system for generating gene knock-out could be applied to some of the other Dictyostelia species.

## Methods

### Strains and cell culture

We cultured *Dictyostelium discoideum* strains AX2 and AX3 and their transformants at 22 °C on culture dishes or in shaking culture in HL5 medium or on SM agar plates with *Klebsiella planticola*. Transformants were maintained in HL5 at 20 µg/ml G418, 50 µg/ml hygromycin B or 10 µg/ml blasticidin S, as appropriate.

### Plasmid constructs

For the construction of the Cas9-GFP expression vector, open reading frame encoding Cas9-NLS was amplified by PCR from pSLQ1658-dCas9-EGFP^22^ (obtained from Addgene). To increase the translation efficiency, we inserted the actin Kozak sequence ATACAACAATAAA^38^ upstream of the Cas9 start codon, and optimised the initial 47 amino acid sequences of Cas9 for *Dictyostelium* codon usage. We attached GFP obtained from the MS2-GFP expression vector^39^ to the C-terminal region in order to monitor the expression and localisation occurring within a single cell. PCR products were then inserted into the pEXP4 (+) vector^40^ via the BglII and SpeI restriction sites, creating the dCas9-NLS-GFP expression vector pTM809. Although the plasmid initially contained the dCas9 sequence, which lacks endonuclease activity, we obtained the Cas9 sequence by adding point mutation in the primers. We joined the fragments together using BpiI so that the resulting Cas9-NLS-GFP vector, pTM1191, was ligated without the addition of extra amino acids (Supplementary Fig. S1A).

To drive the CRISPR target and scaffold sequence of the sgRNA by an RNA polymerase III promoter, we used both the U6 (DDB_G0295505) promoter sequence, amplified by PCR, and the isoleucine tRNA (DDB_G0295381) region, including an artificially synthesised promoter sequence. We cloned a plasmid containing a sgRNA scaffold under the control of a U6 or tRNA promoter into the pGEM-T Easy vector (Supplementary Fig. S1B). These vectors contained two BpiI sites between the promoter and the sgRNA scaffold, and we therefore used the Golden Gate digestion-ligation reaction to insert a pair of annealed oligonucleotides into the 5′ end of the sgRNA scaffold. We selected guide sequences targeting the gene of interest using Cas-Designer^30,41^. To obtain a stable expression vector for sgRNA, we then cloned the promoter and sgRNA into the pDM358 vector digested with XhoI and HindIII.

We generated the all-in-one sgRNA and Cas9 expression vector, pTM1285, via the ligation of 3 fragments, Cas9-NLS-GFP, the sgRNA scaffold and the G418 resistance cassette, and cloned the resulting fragment into a pBlueScript II vector. Thereafter, we cloned a pair of annealed oligonucleotides into the pTM1285 vector pre-digested with BpiI. For generating multiplex sgRNA vectors, we assembled multiple sgRNA expression cassettes using the three-step Golden Gate cloning method. This strategy relies on a Type IIS restriction enzyme that cleaves outside of the recognition site. We generated entry vectors (pEV) used for the first step of the Golden Gate cloning method by inverse PCR using a Platinum Gate TALEN kit as a template^42^. Each vector contained a cassette for sgRNA under the control of tRNA expression. The first step was to clone individual sgRNA into these entry vectors by BpiI digestion and ligation. For the second step, we combined the first four modules in the second module vectors (pFUS) obtained from the Platinum Gate TALEN kit. Eventually, we combined multiple tRNA-sgRNA modules into the final destination vector, pTM1290.

### Transformation and identification of transformants

We harvested cells during the exponential phase of growth, washed them twice in ice-cold H50 buffer (20 mM HEPES, pH 7.0, 50 mM KCl, 10 mM NaCl, 1 mM MgSO_4_, 5 mM NaHCO_3_ and 1 mM NaH_2_PO_4_), and re-suspended them in H50 at a concentration of 5.0 × 10^7^ cells/ml. After a 5-min incubation of plasmid DNA (1–10 µg in 5 µl) and 100 µl of cells on ice, we transferred them to a cold 1-mm electroporation cuvette. Electroporation was performed using a Gene Pulser Xcell system (Bio-Rad) at 0.85 kV twice with a 5 s interval. After 5 min of incubation on ice, we transferred the cells to a culture dish containing 10 ml of HL5. To obtain cells transiently expressing Cas9 and sgRNA, we replaced the medium with HL5 containing 10 µg/ml of G418 after 8–16 h, and cultured the cells for another 3 days. Moreover, we plated the cells on SM agar plates with *K. planticola* and incubated them for 3–4 days until plaque formation. We transferred the individual plaques to 96-well plates containing HL5 in the absence of G418 and incubated them for 2 days until the colonies grew. To select the cell lines sensitive to G418, we transferred 10 µl of aliquots from each well to new 96-well plates containing 150 µl of HL5 with 10 µg/ml of G418. To obtain stably expressing clones, we cultured cells in HL5 containing 20 µg/ml of G418 or 50 µg/ml of hygromycin B for >1 week until the colonies grew, and plated the cells on SM agar plates.

To isolate genomic DNA from the mutants, we re-suspended the cell pellet in lysis buffer (1 × PCR buffer, 1% NP40 and 50 µg/ml of Proteinase K) and incubated the suspension at 56 °C for 45 min. Thereafter, we brought the mixtures to 95 °C for 10 min to inactivate the ProK. We used the cell lysate as a template for PCR. We amplified the CRISPR target site by a 2-step PCR using KOD plus Neo polymerase (TOYOBO), where DNA was denatured at 95 °C for 10 s and annealed/extended at 64–68 °C for 30–60 s. As we had designed one of the PCR primers to span the Cas9 cleavage site, mutation detective PCR showed no amplification in most of the mutated clones. However, the possibility that PCR amplification may be observed in mutated clones cannot be eliminated. For sequence analysis of the targeted regions, we amplified the expected mutation site and cloned it into a pGEM-T Easy vector by TA cloning. For each gene, 4 independent clones were sequenced. The primers used here are listed in Supplementary Table S1. For mutation detective PCR, the target and Screening-Rv primers were used. For sequencing, the targeted region was amplified with primers Screening-Rv and Screening-Fw.

### Calculation of targeting efficiency

We generated a tdTomato knock-in cell line by knocking in the tdTomato-floxed-bsr to the *act5* gene locus and removing the bsr cassette using the Cre-*loxP* system. We quantified the efficiency of targeting by monitoring the decrease in fluorescence within a single cell. We stained the cells with 10 µM of CellTracker Green CMFDA (Life Technologies) for 30–60 min, and then washed them twice with KK2 (20 mM potassium phosphate, pH 6.2). We acquired green and red fluorescence images of the cells using an Olympus IX71 inverted fluorescence microscope with a 1.35 NA 60 × objective and Orca-Flash4.0 V2 Digital CMOS camera (Hamamatsu). We calculated the intensity of tdTomato by averaging the intensity within each cell (green region). In the situation where the intensity was similar to that of the wild type (AX3), the cell was defined as a targeted cell. We performed image processing and analysis with ImageJ and Volocity (Perkin Elmer).

Since the disruption of both *acaA* and *pkaC* reveal aggregation deficiency during development, we assessed the CRISPR-mediated gene targeting by analysing the aggregation phenotype. We plated each clone on SM plates with a bacterial lawn and incubated them for 3–4 days until large plaques were formed. We calculated the targeting efficiency by counting the number of aggregation-deficient clones.

### Analysis of RNA

Total RNA, including small RNAs, was isolated from dCas9-GFP and sgRNA expressing cell lines using RNAiso Plus (Takara), according to the manufacturer’s instruction. We treated the purified RNA samples with DNaseI to minimise DNA contamination. For circular RT-PCR (cRT-PCR), we circularised the RNA using T4 RNA ligase and reverse transcribed it using sgRNA specific primer (sgRNA-Rv), CCAGCATAGCTCTTAAACCCGTCTTC, as described previously^10^. We amplified the circularised DNA using the primer pair of sgRNA-Rv and sgRNA-Fw, AAATAAGGCTAGTCCGTTATCAACTTGAAAAAG. For conventional RT-PCR, we used random primers to synthesise first strand DNA, and amplified the DNA using specific primers. DNA gels were imaged using FAS-V imaging system.

## Electronic supplementary material

Supplementary Information
