# Supplementary material for: CRISPR/Cas9 mediated targeting of multiple genes in Dictyostelium
Source: Sci Rep. 2018 May 31;8:8471. doi: 10.1038/s41598-018-26756-z (PMC5981456; doi:10.1038/s41598-018-26756-z)
Supplement: Supplementary file 1 — Supplementary Information [file 41598_2018_26756_MOESM1_ESM.pdf]

CRISPR/Cas9 mediated targeting of multiple genes in *Dictyostelium*

Ryoya Sekine, Takefumi Kawata, Tetsuya Muramoto*

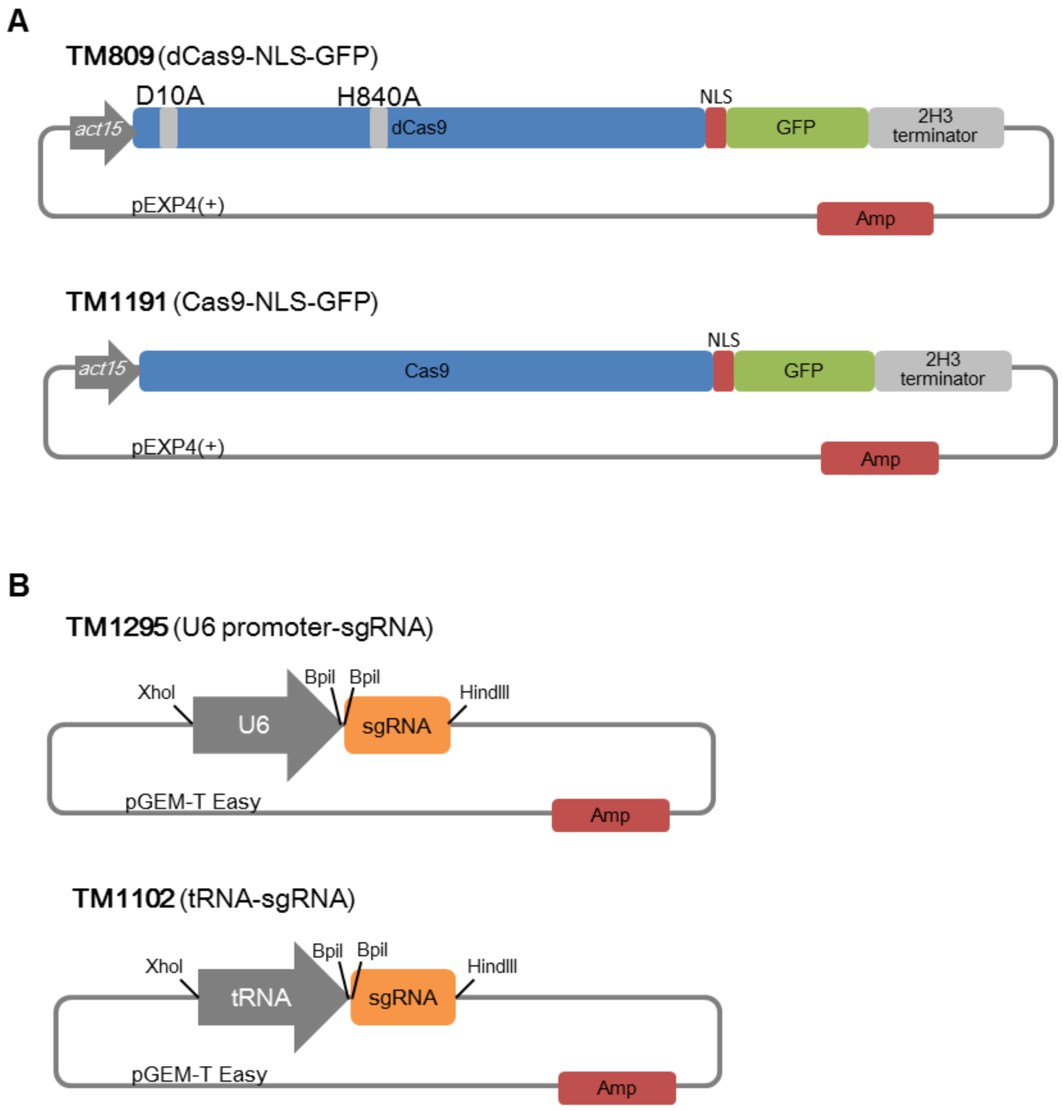

Supplementary Figure S1. Optimised Cas9 expression vectors in *Dictyostelium*.

(A) The dCas9 and Cas9 expression vectors modified from the previous vector system, pSLQ1658-dCas9-EGFP. (B) An sgRNA expression vector under the control of U6 or tRNA. U6, U6 promoter; tRNA, isoleucine tRNA.

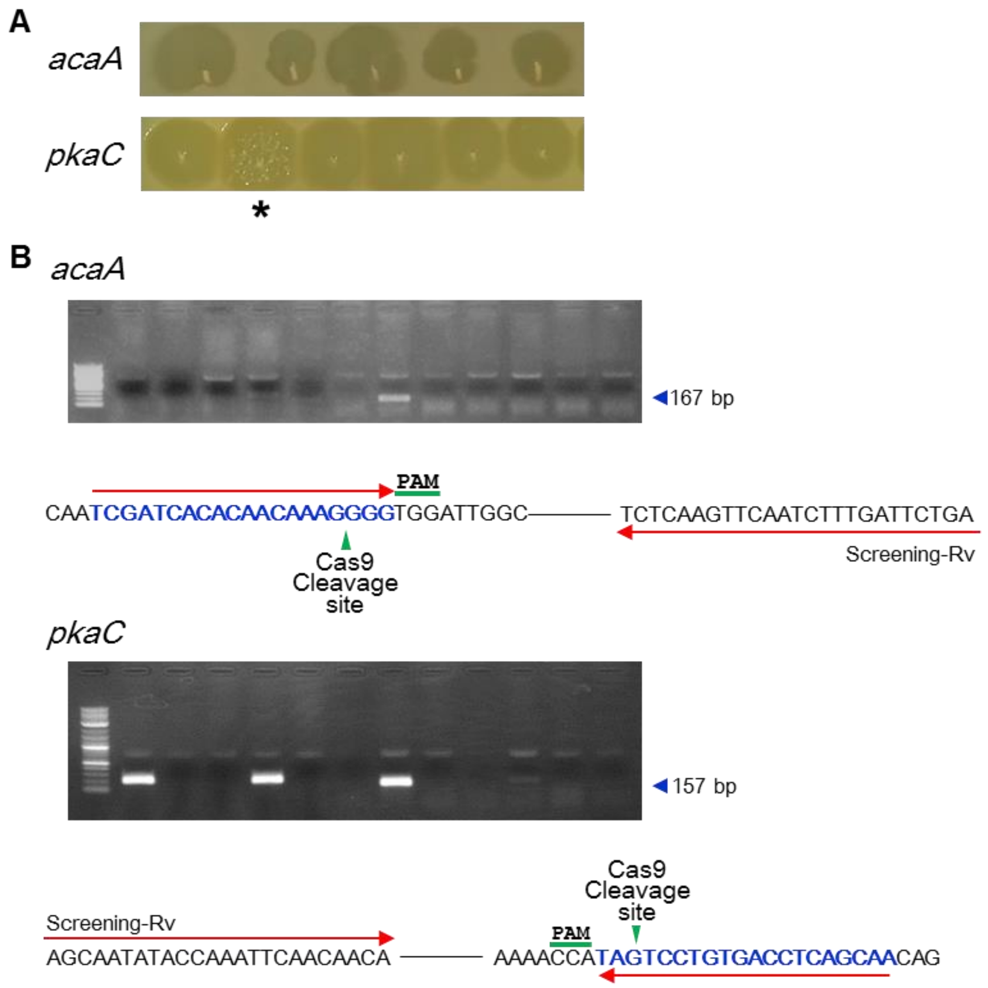

Supplementary Figure S2. Analysis of *acaA* and *pkaC* mutants.

(A) Aggregation phenotype of *acaA* and *pkaC* mutants. Single clones were seeded on a bacteria lawn on SM agar plates and imaged after 3-4 days. The asterisk indicates aggregation-positive clone and rest of the clones are aggregation-negative. (B) PCR analysis of individual aggregation-negative clones. The positions of PCR primers relative to the Cas9 cleavage site are presented by red arrows. One of the primers was designed to span the cleavage site, thus no PCR amplifications is expected in mutated clones. Blue arrowheads indicate PCR products observed with wild type cells. The size of the PCR products are 167 bp and 157 bp, respectively. Gel images were cropped, but no other bands were present.

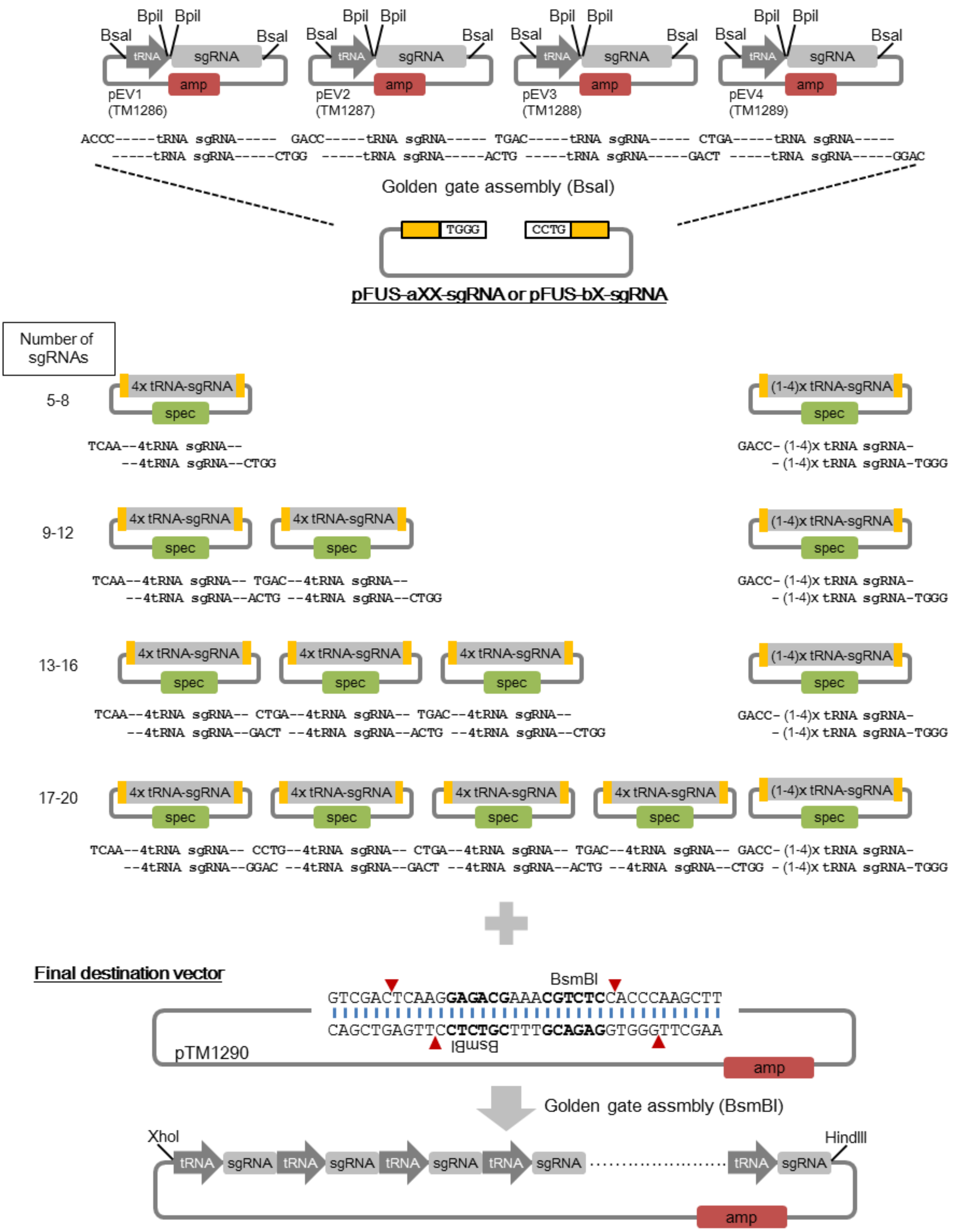

Supplementary Figure S3. Schematic diagram to construct multiplex sgRNA expression vector using Golden Gate assembly.

The target sequence of the individual locus was synthesised and inserted into the Golden Gate entry

vectors (pEV) digested by BpiI. The cloned fragment in the pEV is flanked by BsaI sites and can be sub-cloned into the second module vectors (pFUS) obtained from the Platinum Gate TALEN kit. Once the desired numbers of fragments are constructed, second steps of Golden Gate assembly are performed by BsmBI digestion and ligation step.

Supplementary Table S1. Primer sequences used for cloning and sequencing

Gene	Description	Sequence
tdTomato	sgRNA sense	AGCAATGTCCCAGGCGAAGGGCAG
tdTomato	sgRNA antisense	AAACCTGCCCTTCGCCTGGGACAT
tdTomato	mutation detection Fw	TTGTTGGTTGCTTTATTTTATTTGA
tdTomato	mutation detection Rv	AGCAAATCAAAATAATCTGGAAGT
acaA	sgRNA sense	AGCATCGATCACACAACAAAGGGG
acaA	sgRNA antisense	AAACCCCCTTTGTTGTGTGATCGA
acaA	Screening-Rv	TCAGAATCAAAGATTGAACTTGAGA
acaA	Screening-Fw	GGTGGTCATATTAATCCCATGC
pkaC	sgRNA sense	AGCATTGCTGAGGTCACAGGACTA
pkaC	sgRNA antisense	AAACTAGTCCTGTGACCTCAGCAA
pkaC	Screening-Rv	AGCAATATACCAAATTCAACAACA
pkaC	Screening-Fw	CTGGCCAAGATATGAAATAGAGG
pikA	sgRNA sense	AGCAAGTGGAGGACATGTAAATAA
pikA	sgRNA antisense	AAACTTATTTACATGTCCTCCACT
pikA	Screening-Rv	GCAGATTCATCACTACCACCA
pikA	Screening-Fw	ACCCACCCACAACAGATGA
pikB	sgRNA sense	AGCAAGTTCAACATTGATGTACAA
pikB	sgRNA antisense	AAACTTGTACATCAATGTTGAACT
pikB	Screening-Rv	TTCAATTGCTGGTGGTGGTA
pikB	Screening-Fw	GTGGTGGTAGTAACCTCAGTGTAAA
pikC	sgRNA sense	AGCAAAATACAGTTGTGCAAGGTT
pikC	sgRNA antisense	AAACAACCTTGCACAACTGTATTT
pikC	Screening-Rv	TGGTTGTTGGAAGTATTGAGGA
pikC	Screening-Fw	TCAATGGTTGCCTTGACATT
pikF	sgRNA sense	AGCATGAAAAAACAGCTGTTGAAT
pikF	sgRNA antisense	AAACATTCAACAGCTGTTTTTTCA
pikF	Screening-Rv	AAATGAACGTGGTGGTACAGC
pikF	Screening-Fw	CTGAATCAGATGATGAAGGTGA
pikG	sgRNA sense	AGCACATTAAGTTTAGTTTCTAAA
pikG	sgRNA antisense	AAACTTTAGAAACTAAACTTAATG
pikG	Screening-Rv	GAAAAGTCTTATAATTCAAGTTCACCA
pikG	Screening-Fw	CATACTATTTGGTCCACTATTCGTT
